# Pain coping skills training for African Americans with osteoarthritis study: baseline participant characteristics and comparison to prior studies

**DOI:** 10.1186/s12891-018-2249-6

**Published:** 2018-09-19

**Authors:** Kelli D. Allen, Liubov Arbeeva, Crystal W. Cené, Cynthia J. Coffman, Kimberlea F. Grimm, Erin Haley, Francis J. Keefe, Caroline T. Nagle, Eugene Z. Oddone, Tamara J. Somers, Yashika Watkins, Lisa C. Campbell

**Affiliations:** 1 Thurston Arthritis Research Center, University of North Carolina at Chapel Hill, 3300 Thurston Bldg., CB# 7280, Chapel Hill, NC 27599-7280 USA; 2 Department of Medicine, University of North Carolina at Chapel Hill, 125 MacNider Hall CB# 7005, Chapel Hill, NC 27599 USA; 3 Health Services Research and Development Service, Durham VA Medical Center, Durham, NC USA; 4 Department of Biostatistics and Bioinformatics, Duke University Medical Center, Durham, NC USA; 5 Department of Psychology, East Carolina University, Greenville, NC USA; 6 Department of Psychiatry and Behavioral Science, Duke University, Durham, NC USA; 7 Department of Medicine, Duke University Medical Center, Durham, NC USA; 8 Institute for Health Research and Policy, University of Illinois at Chicago, Chicago, IL USA

**Keywords:** Osteoarthritis, Knee, Hip, Pain coping skills training, Health disparities

## Abstract

**Background:**

The Pain Coping **S**kills **T**raining for **A**frican **A**mericans with Osteoa**RT**thritis (STAART) trial is examining the effectiveness of a culturally enhanced pain coping skills training (CST) program for African Americans with osteoarthritis (OA). This disparities-focused trial aimed to reach a population with greater symptom severity and risk factors for poor pain-related outcomes than previous studies. This paper compares characteristics of STAART participants with prior studies of CST or cognitive behavioral therapy (CBT)-informed training in pain coping strategies for OA.

**Methods:**

A literature search identified 10 prior trials of pain CST or CBT-informed pain coping training among individuals with OA. We descriptively compared characteristics of STAART participants with other studies, in 3 domains of the National Institutes of Minority Health and Health Disparities’ Research Framework: Sociocultural Environment (e.g., age, education, marital status), Biological Vulnerability and Mechanisms (e.g, pain and function, body mass index), and Health Behaviors and Coping (e.g., pain catastrophizing). Means and standard deviations (SDs) or proportions were calculated for STAART participants and extracted from published manuscripts for comparator studies.

**Results:**

The mean age of STAART participants, 59 years (SD = 10.3), was lower than 9 of 10 comparator studies; the proportion of individuals with some education beyond high school, 75%, was comparable to comparator studies (61–86%); and the proportion of individuals who are married or living with a partner, 42%, was lower than comparator studies (62–66%). Comparator studies had less than about 1/3 African American participants. Mean scores on the Western Ontario and McMaster Universities Osteoarthritis Index pain and function scales were higher (worse) for STAART participants than for other studies, and mean body mass index of STAART participants, 35.2 kg/m^2^ (SD = 8.2), was higher than all other studies (30–34 kg/m^2^). STAART participants’ mean score on the Pain Catastrophizing scale, 19.8 (SD = 12.3), was higher (worse) than other studies reporting this measure (7–17).

**Conclusions:**

Compared with prior studies with predominantly white samples, STAART participants have worse pain and function and more risk factors for negative pain-related outcomes across several domains. Given STAART participants’ high mean pain catastrophizing scores, this sample may particularly benefit from the CST intervention approach.

**Trial registration:**

NCT02560922

**Electronic supplementary material:**

The online version of this article (10.1186/s12891-018-2249-6) contains supplementary material, which is available to authorized users.

## Background

African Americans bear a disproportionate burden of chronic pain conditions, including osteoarthritis (OA). Compared with Caucasians, African Americans not only have a higher prevalence of OA, but also more severe pain, functional limitations, and other negative outcomes [1–6]. Because of these well-documented racial differences, the Institute of Medicine has identified interventions to reduce disparities in OA and other musculoskeletal diseases among its top 25 (highest tier) priority topics for comparative effectiveness research [7]. In alignment with this priority, the Pain Coping **S**kills **T**raining for **A**frican **A**mericans with Osteoa**RT**thritis (STAART) study is evaluating the effectiveness of a culturally enhanced pain coping skills training (CST) program for African Americans with OA [8]. There were several factors underlying the choice of a pain CST intervention to address racial disparities in OA-related pain and other outcomes. First, when compared with Caucasians, African Americans with chronic pain conditions report greater levels of pain catastrophizing (i.e., the tendency to focus on and magnify pain sensations and to feel helpless in the face of pain [9–13]), lower perceived ability to cope with and control pain [14], and greater maladaptive coping strategies (i.e., emotion-focused or external coping strategies) [4, 10, 14–16]. These coping-related characteristics have been associated with worse pain, function, and depressive symptoms [17–19]. Second, previous studies indicated that pain coping and other psychological factors are key factors underlying racial differences in OA-related pain [3, 4]. Third, pain CST interventions have been shown to enhance and improve coping strategies, OA-related pain and other outcomes [20–25]. However, there has been very limited study of pain CST interventions among African Americans with OA or other musculoskeletal conditions. This is important because of evidence that behavioral and psychological interventions are most effective when they are adapted to meet the needs and expectations of minority populations [26].

Based on prior studies of racial differences in pain, coping, and social determinants of health [3, 11–16, 27, 28], we expected that baseline characteristics of the STAART study participants, who are all African American, would reflect a worse risk profile than those of participants in prior studies of pain CST or other cognitive behavioral therapy (CBT)-informed training in pain coping strategies. Therefore, the objective of this analysis was to descriptively compare characteristics of STAART study participants with prior studies of pain CST or CBT-informed pain coping strategies among individuals with OA. In particular, we focused on Individual Level Sociocultural Environment, Biological and Behavioral domains, within the National Institute on Minority Health and Health Disparities (NIMHD) Research Framework [29] as these are of highest relevance to this intervention and population. This Framework also has domains at the Interpersonal Level (e.g., family functioning, patient-clinician relationship), Community Level (e.g. community resources, availability of health services), and Societal Level (e.g., policies and laws); although some items in these domains are also relevant to this intervention and patient group, variables within these domains were not assessed in STAART.

## Methods

### Overview of STAART study

The STAART study, described in detail previously [8], is a randomized controlled trial of 248 African Americans with symptomatic hip or knee OA. The STAART study enrolled only African Americans (vs inclusion of other racial groups) so that in-depth efforts could be focused on this demographic group with a high risk for poorer OA and pain-related outcomes. STAART participants are equally allocated to pain CST and wait list control groups. The pain CST intervention involved 11 phone-based sessions, delivered approximately weekly and based on previous pain CST programs [20, 22, 23, 25, 30]. Participants in the wait list group received only their usual care for OA, with no study intervention offered until after completion of final follow-up assessments. All measures for these analyses were collected from patients prior to their randomization to treatment conditions. This study was approved by the Institutional Review Boards of the University of North Carolina (UNC), the Durham VA Healthcare System (VA), Duke University Medical Center and East Carolina University.

### STAART participants and recruitment methods

Participants were recruited from the UNC Healthcare System and the Durham VA; 124 participants were enrolled from each site. Study inclusion were 1) Diagnosis of knee or hip OA, verified by self-reported diagnosis from a medical professional, including items based on the American College of Rheumatology clinical criteria for knee or hip OA, 2) Self-report of pain, aching, or stiffness in one or both knees or hips on most days of the week, 3) Patient of the UNC Health Care System or Durham VAMC. Exclusion criteria have been described previously [8] and generally include other pain-related conditions that confound study outcomes or health conditions that would prevent participation in the intervention (e.g., severe hearing loss since this was a phone-based intervention).

Three general methods of recruitment were used. First, potentially eligible patients were identified from UNC and VA medical records, based on OA diagnosis codes; these patients were mailed letters inviting participation, followed by a telephone call. Second, advertisements were posted at study sites and surrounding communities, inviting patients to self-refer to the study. Third, health care providers at study sites could refer patients to the study team directly, with patients’ permission, or give study brochures to patients. We used an enhanced informed consent process that included education about the research process, participant bill of rights, and perspectives from African Americans who have participated in research [31].

### Measures

The following measures, representing three Individual Level domains from the NIMHD Research Framework, were assessed in-person at baseline by a trained research assistant. Some of these measures are reported for the STAART study sample only (Table 1) because they were not available for any comparator studies, but they represent key constructs related to health disparities and the NIMHD Framework.

**Table 1:** Characteristics of STAART Participants, Overall and By Site

Characteristic	Total Sample Mean ± SD or N(%)	DVAHCS Mean ± SD or N(%)	UNC Mean ± SD or N(%)
Sociocultural Environment			
Age	59.0 ± 10.3	57.8 ± 10.0	60.2 ± 10.5
Female	122 (49.2%)	26 (21.0%)	96 (77.4%)
Hispanic	7 (2.9%)	2 (1.6%)	5 (4.3%)
Some education above high school	187 (75.4%)	101 (81.5%)	86 (69.4%)
Working	86 (34.7%)	47 (37.9%)	39 (31.5%)
Married or living with partner	103 (41.5%)	63 (50.8%)	40 (32.3%)
Low Perceived Income^a^	83 (33.6%)	39 (31.7%)	44 (35.5%)
Duke University Religion Index			
Attendance at Religious Activities	4.4 ± 1.4	4.1 ± 1.5	4.8 ± 1.2
Private Religious Activities	4.0 ± 1.6	3.8 ± 1.6	4.2 ± 1.5
Intrinsic Religiosity	13.1 ± 2.5	13.1 ± 2.4	13.2 ± 2.6
Biological Vulnerability and Mechanisms			
WOMAC Total^b^	53.0 ± 17.8	56.0 ± 16.6	49.9 ± 18.5
WOMAC Pain	11.0 ± 3.9	11.6 ± 3.8	10.4 ± 3.9
WOMAC Function	37.0 ± 13.2	39.1 ± 12.4	34.9 ± 13.7
PROMIS Pain Interference Score	63.8 ± 6.9	64.0 ± 6.3	63.5 ± 7.5
Short Form-12 - Mental	50.7 ± 11.1	49.8 ± 11.6	51.5 ± 10.7
Short Form-12 - Physical	33.1 ± 9.1	33.1 ± 8.4	33.1 ± 9.7
Duration of Arthritis Symptoms	13.1 ± 10.0	14.6 ± 10.6	11.5 ± 9.1
Number of Self-Reported Comorbidities	8.5 ± 3.9	8.2 ± 3.5	8.8 ± 4.3
Body Mass Index (kg/m^2^)	35.2 ± 8.2	33.1 ± 6.5	37.5 ± 9.1
Health Behaviors & Coping Strategies			
CSQ – Total Coping Attempts	93.9 ± 36.6	91.9 ± 39.7	95.8 ± 33.3
CSQ – Diverting Attention	13.9 ± 8.4	12.9 ± 8.6	14.8 ± 8.2
CSQ – Ignoring Sensations	14.1 ± 8.7	15.0 ± 9.6	13.2 ± 7.7
CSQ – Coping Self-Statements	23.6 ± 7.6	23.4 ± 8.2	23.7 ± 6.8
CSQ – Reinterpreting Pain Sensations	8.9 ± 8.4	9.7 ± 8.8	8.1 ± 8.0
CSQ – Praying, Hoping	20.8 ± 8.0	19.8 ± 8.0	21.9 ± 7.9
CSQ – Increasing Behavioral Activities	12.5 ± 7.1	11.0 ± 7.0	14.0 ± 6.9
CSQ – Catastrophizing	11.4 ± 7.6	11.1 ± 7.3	11.7 ± 7.8
Pain Catastrophizing Scale	19.8 ± 12.3	20.4 ± 12.4	19.2 ± 12.1
PHQ-8	6.2 ± 5.3	6.6 ± 5.3	5.7 ± 5.3
Arthritis Self-Efficacy Scale	5.9 ± 2.0	5.6 ± 2.1	6.1 ± 1.8
Self-Efficacy for Pain Communication	78.7 ± 22.0	77.4 ± 22.9	80.1 ± 21.1
Brief Fear of Movement Scale	14.8 (3.5)	14.4 (3.9)	15.2 (3.1)

*DVAHCS* Durham VA Health Care System, *UNC* University of North Carolina, *WOMAC* Western Ontario and McMaster Universities Osteoarthritis Index, *CSQ* coping strategies questionnaire, *PHQ* patient health questionnaire

^a^ Self-report of “just meet basic expenses” or “don’t even have enough to meet basic expenses.”

^b^Likert scale version of WOMAC

#### Sociodemographics (individual level sociocultural environment domain)

##### Age

Participant age was based on date of birth from the electronic medical record and confirmed through self-report.

##### Sex

Participant sex was based on the electronic medical record and confirmed through self-report.

##### Ethnicity

Participants self-reported whether they were of Hispanic / Latino descent or not.

##### Education

Participants selected from eight options ranging from grade school/junior high to post graduate work or graduate degree. For these analyses we grouped individuals as having “above high school education” or not, as this was the most common categorization that could be ascertained from comparator studies.

##### Work status

Participants selected from seven options regarding work status, and for these analyses individuals were grouped as either working or not at the time of the study.

##### Household financial status

Participants selected from four options regarding their household’s financial situation and were grouped as either “live comfortably” or “meet basic expenses with a little left over for extras” vs. “just meet basic expenses” or “don’t even have enough to meet basic expenses.”

##### Marital status

Participants selected from five options regarding current marital status and for these analyses were grouped as being married / living with a partner as married or not currently married at the time of the study.

##### Religiosity

This measure was included in STAART because of its cultural relevance in the African American community. The Duke University Religion Index (DUREL) is a five-item measure of religious beliefs and experience [32]. The index consists of 3 subscales, recording the frequency of attendance at religious services (subscale 1; range 1–6), the frequency of private religious activities (subscale 2; range 1–6) and intrinsic religiosity (subscale 3; range 3–15). Higher scores represent more religious activities or religiosity.

#### Biological vulnerability and mechanisms (individual level biological domain)

##### Pain and function - Western Ontario and McMaster Universities Osteoarthritis Index (WOMAC)

The WOMAC is a measure of lower extremity pain (5 items), stiffness (2 items), and function (17 items) [33, 34]. All items were rated on a Likert scale of 0 (no symptoms) to 4 (extreme symptoms), with a total range of 0–96 and higher scores indicating worse symptoms. Pain and function subscales are also separately reported. Some other studies used the Visual Analog Scale (VAS) version of the WOMAC, which includes the same items but each measured on a 100 mm VAS. For this version, each subscale score ranges from 0 to 100, with higher scores indicating worse symptoms or function. To facilitate comparison of STAART with studies using the WOMAC VAS version, we also transformed pain and function domains to a 0–100 scale, which has been done in prior studies [35, 36].

##### Arthritis impact measurement scales (AIMS)

The AIMS was not collected in the STAART study. However, it is a common measure in other OA studies, and therefore we present it for comparator studies when available. Although this does not allow a direct comparison to WOMAC, the AIMS scale provides a general description of the symptom severity of participants in comparator studies. Comparator studies used both the original AIMS, the AIMS2 and the AIMS2 Short Form (AIMS2-SF). The original AIMS includes 45 items across the domains of pain, physical disability and psychological disability [37]; the latter 2 are reported here because of their similarity to WOMAC domains. Each AIMS domain has a score range of 0–10, with higher scores indicating greater pain or disability. The AIMS2 is an expanded version with 78 items, and the AIMS2-SF has 26 items [38, 39]. AIMS2 domains also have score ranges of 0–10, with higher scores indicating greater pain or disability.

##### PROMIS pain interference instrument (short form)

The PROMIS Pain Interference (Short Form 6a) instrument measures the self-reported consequences of pain across aspects of life including social, cognitive, emotional, physical and recreational activities; this instrument refers to the past 7 days [40] This validated scale has five response options, with scores ranging from one to five; items are summed and re-scaled as a t-score with mean of 50 and standard deviation of 10.

##### Health-related quality of life (HRQoL) – Short form 12 (SF-12)

The Short-Form-12 (SF-12) is a validated measure that covers domains of general health, physical health, work and activity limitations, and emotional health [41]. Physical and Mental Health Composite Scores (PCS & MCS) are computed using the scores of 12 questions and range from 0 to 100, with higher scores indicating better health.

##### Duration of arthritis symptoms

Participants self-reported the number of years they have been experiencing knee and / or hip arthritis symptoms (joint pain, stiffness, or limited movement).

##### Comorbid illnesses

The Self-Administered Comorbidity Questionnaire asks participants to indicate whether or not they have each of 13 physical and psychological health conditions. Participants can also list up to 3 additional conditions. The score range is 0–16 [42].

##### Body mass index (BMI)

BMI was calculated from measured height and weight at baseline.

#### Health behaviors & coping strategies (individual level behavioral domain)

##### Coping strategies questionnaire (CSQ)

The CSQ is the most commonly used measure of coping among individuals with chronic pain, and its measurement properties have been confirmed in patients with a variety of pain-related conditions [43, 44]. This scale includes 48 items that assess 6 cognitive domains (Catastrophizing, Diverting Attention, Ignoring Sensations, Coping Self-Statements, Reinterpreting Pain Sensations, Praying-Hoping) and 1 behavioral domain (Increasing Behavioral Activities). Each domain includes 6 items, and participants rate the frequency of their use of specific coping strategies on a 7-point Likert scale from 0 (“Never do that”) to 6 (“Always do that”). From the CSQ, we calculated a Coping Attempts Score, which sums all domains other than Catastrophizing. This score was reported because it could be compared to other previous studies [45, 46], and because the factor structure for this score has been replicated in prior research [47–49]. We also separately report scores for the Catastrophizing subscale.

##### Pain catastrophizing scale (PCS)

The PCS is a widely used measure of catastrophic thinking related to pain [50]. This 13-item instrument asks participants to reflect on past painful experiences and to indicate the degree to which they experienced each of the thoughts or feelings when experiencing pain, with each item scored from 0 (not at all) to 4 (all the time). The PCS includes 3 subscales – rumination, magnification, and helplessness. Scores for all items are summed and total scores range from 0 to 52 with a higher score indicating a higher level of catastrophizing.

##### Depressive symptoms – Patient health questionnaire 8 (PHQ-8)

The PHQ-8 is an eight-item survey that consists of items corresponding to depression criteria listed in the *Diagnostic and Statistics Manual Fourth Edition* (DSM-IV) [51]. Each of the eight questions is scored as 0 (not at all) to 3 (nearly every day), so that total scores range from 0 to 24.

##### Arthritis self efficacy scale

This scale includes 8 items asking respondents how certain they are that they can perform specific activities or tasks [52]. Items are scored on a Likert Scale (1 = very uncertain to 10 = very certain); the total score represents a mean of the 8-items, with a range of 1–10. Because of challenges with comparing scores across different versions of this scale, we only included comparator studies that used the 8-item version.

##### Self-efficacy for pain communication scale – Patient version [53]

This 7-item instrument assesses patient’s level of confidence in communicating their pain to a “significant other” and receiving understanding and a helpful response. Items are rated on a scale from 10 (“very uncertain”) to 100 (“very certain”).

##### Brief fear of movement scale

The Brief Fear of Movement Scale is a six item scale for assessing fear of movement in OA [54]. All items are measured on a 4-point scale from “strongly agree” to “strongly disagree.” The total score ranges from 6 to 24, with higher scores indicating more fear of movement.

### Comparator studies

We aimed to identify prior studies of pain CST and CBT-informed pain coping strategies among individuals with OA (regardless of racial composition), since those are of highest relevance to the STAART study. To identify comparator studies, we performed a literature search (using Pubmed) with search terms of (osteoarthritis) AND (CST OR CBT). We included clinical trials meeting these criteria from any country, resulting in 10 studies. We also compared our identified studies to a recent systematic review of behavioral intervention for OA and found no additional studies to include. For each of study we extracted relevant baseline participant characteristics for comparison to STAART. When participant characteristics were presented only by treatment arm, we contacted authors to request characteristics for the full study sample for simplicity of comparisons. When these were not available we presented characteristics by treatment arm. We compared characteristics between STAART participants and other studies descriptively. Because of the relatively small number of studies and because not all studies assessed all measures of interest, we did not conduct statistical comparisons.

The following are summaries of the studies we identified and included in this comparison. Additional details on participant inclusion criteria and recruitment methods are reported in Additional file 1:

**Effectiveness of an Internet-Delivered Exercise and Pain-Coping Skills Training Intervention for Persons With Chronic Knee Pain: A Randomized Trial (Bennell et al., 2017)** [45].

- Participants: 148 patients with knee pain.
- Intervention: online educational materials, an interactive, automated 8-module pain CST program (PainCOACH), and seven Skype sessions with a physiotherapist for 12 weeks, focusing on a home exercise program.
- Comparator group: online educational materials only.

**Physical Therapist-Delivered Pain Coping Skills Training and Exercise for Knee Osteoarthritis: Randomized Controlled Trial (Bennell et al., 2016)** [55, 56].

- Participants: 222 patients with symptomatic knee OA.
- Interventions: pain CST only, exercise only or pain CST/exercise combined. All groups attended 10 individual sessions with a physical therapist for 12 weeks, plus a home program.

**Automated Internet-based pain coping skills training to manage osteoarthritis pain: a randomized controlled trial (Rini et al., 2015)** [57].

- Participants: 113 participants with hip or knee OA
- Interventions: Internet-based PCST (PainCOACH), eight modules in a self-directed manner at a rate of one per week
- Comparator group: assessment-only control group

**Nurse practitioners can effectively deliver pain coping skills training to osteoarthritis patients with chronic pain: A randomized, controlled trial (Broderick et al., 2014)** [58].

- Participants: 256 participants with symptomatic knee or hip OA
- Intervention: 10 individual weekly sessions of pain CST
- Comparator group: usual care

**Effectiveness of a cognitive-behavioural group intervention for knee osteoarthritis pain: a randomized controlled trial (Helminen et al., 2014)** [59].

- Participants: 111 patients with symptomatic knee OA
- Intervention: CBT program for pain management, delivered in 6 weekly group sessions led by both a psychologist and a physiotherapist
- Comparator group: regular general practitioner care only

**Cognitive-behavioral treatment for comorbid insomnia and osteoarthritis pain in primary care: the lifestyles randomized controlled trial (Vitiello et al., 2013)** [60].

- Participants: 367 individuals with symptomatic OA and insomnia,
- Interventions: CBT for pain and insomnia, CBT for pain or education. CBT interventions were delivered in groups at primary care clinics and consisted of 6 weekly 90-min sessions.
- Comparator group: usual care

**Pain coping skills training and lifestyle behavioral weight management in patients with knee osteoarthritis: a randomized controlled study (Somers et al., 2012)** [25].

- Participants: 232 individuals with symptomatic knee OA
- Interventions: pain CST plus lifestyle behavioral weight management (BWM), pain CST only, BMW only. Pain CST only and BWM only interventions had 12 weekly 60-min sessions, followed by bi-weekly 60-min sessions for 12 weeks. The BWM only group also had three weekly supervised sessions weekly for the first 12 weeks. The pain CST + BWM group had 12 weekly 120 min sessions, in addition to 3 weekly supervised exercise sessions, followed by bi-weekly 120-min sessions for 12 weeks.
- Comparator group: standard care

**Clinical effectiveness of a rehabilitation program integrating exercise, self-management, and active coping strategies for chronic knee pain: a cluster randomized trial (Hurley et al., 2007)** [61].

- Participants: 418 individuals with knee pain.
- Interventions: individual rehabilitation, group rehabilitation (8 patients per group). Both individual and group rehabilitation involved 12 sessions (twice weekly for 6 weeks), supervised by a physiotherapist. Content included instruction in pain coping and self-management, as well as an individualized progressive exercise program.
- Comparator group: usual care

**Spouse-assisted coping skills training in the management of osteoarthritic knee pain (Keefe et al., 1996)** [46].

- Participants: 88 married persons with knee OA
- Interventions: spouse assisted pain CST, conventional pain CST with no spouse involvement arthritis education-spousal support control. Participants in all three interventions met in groups of 4 to 6 patients (or couples) for 10 weekly, 2-h group sessions.

**Pain coping skills training in the management of osteoarthritic knee pain: A comparative study (Keefe et al., 1990)** [20, 62].

- Participants: 99 patients with knee OA
- Interventions: pain CST, arthritis education. Both interventions met in small groups (6 to 9 patients) for 10 weekly 90-min sessions.
- Comparator: standard care control

## Results

At both STAART study sites (UNC, Durham VA), 124 participants were enrolled. At UNC, 381 participants refused and 123 were ineligible; at the Durham VA, 632 participants refused and 77 were ineligible. At UNC, the mean ages for consented, refused, and ineligible patients, respectively, were: 60.2 (standard deviation (SD) = 10.5), 64.0 (SD = 12.9), and 60.1 (SD = 12.7); the proportions of females among those consented, refused, and ineligible, respectively, were: 77, 69 and 70%. At the VA, the mean ages for consented, refused, and ineligible patients, respectively were: 57.8 (SD = 10.0), 59.9 (SD = 11.3), and 61.2 (SD = 11.1); the proportions of females among those consented, refused, and ineligible, respectively, were: 21.0, 11.7 and 18.2%. Characteristics of consented STAART participants, overall and by site, are shown in Table 1. Table 2 compares Sociocultural Environment, Biological Vulnerability and Mechanisms, and Health Behaviors and Coping variables for STAART participants and comparator studies; this table includes variables for which there was at least one comparator study that included the measure.

**Table 2:** Characteristics of participants in STAART and comparator studies of pain CST and CBT for patients with osteoarthritis

Participant Characteristic	STAART	Bennell et al, 2017 [45]	Bennell et al, 2016 [55]	Rini et al, 2015 [57]	Broderick et al, 2014 [58]	Helminen, 2014 [59]	Vitiello, 2013 [60]	Somers et al, 2012 [25]	Hurley, 2007 [61]	Keefe et al, 1996 [46]	Keefe et al, 1990 [62]
Sociocultural Environment											
Geographic Region	United States (Central North Carolina)	Australia (7 of 8 states)	Australia (Melbourne and Brisbane)	United States (Central North Carolina)	United States (New York, Virginia and North Carolina)	Finland (Kuopio, Eastern Finland)	United States (Washington State)	United States (Central North Carolina)	United Kingdom (Southeast London)	United States (Central North Carolina)	United States (Central North Carolina)
Age^a^	59.0 ± 10.3	61.2 ± 7.1	63.4 ± 8.1	67.6 ± 9.4	67.2 ± 9.5	CBT: 64.5 ± 7.3 Control Group: 62.8 ± 7.2	73.0 ± 8.0	58.0 ± 10.4	Individual Rehab: 66 (50-91) ^f^ Group Rehab:68 (51-84) Control Group: 67 (51-89)	62.6 ± 10.1	64.0 ± 11.5
Female	49%	56%	60%	81%	77%	69%	78%	79%	70%	61%	72%
Race	100% African American	--	--	70% White 29% African American 2% Other	13% Non-white	--	9% Non-white	38% Non-white	--	--	--
Ethnicity	3% Hispanic/Latino	--	--	11% Hispanic/ Latino	--	--		--	--	--	--
Education	75% Above High School	75% Above High School	61% Above High School	71% Above High School	72% Above High School	--	--	86% Above High School	--	--	--
Work Status	35% Currently Working	57% Currently Working	51% Currently Working	21% Currently Working	30% Currently Working	68% Senior High/ Vocational School or more	86% Above High School	--	--	--	--
Marital Status	41% Married/Living with Partner	--	--	62% Married/Living with Partner	63% Married/Living with Partner	21% Currently Working 66% Married/Living with Partner	78% Retired	--	--	--	--
Biological Vulnerability & Mechanisms											
WOMAC Total Score^a^	^b^53.0 ± 17.8 ^c^53.7 ± 19.1	--	--	--	--	--	-		^b^Individual Rehab: 37.5±19.4 Group Rehab: 39.1±19.7 Control Group: 38.6±19.6	--	--
WOMAC Pain Subscale Score^a^	^b^11.0 ± 3.9 ^c^55.0 ± 19.4	^b^ CST + Exercise: 9.0 ± 2.4 Control: 9.2 ± 2.5	^b^ Exercise: 8.6 ± 2.7 CST: 8.7 ± 2.8 CST + Exercise: 9.0 ± 2.8	--	^c^ 54.8 (17.4)	^c^ CBT: 57.6 (53.9−61.3) ^e^Control Group: 56.4 (52.9−60.0)	--	^c^CST+Weight Management (WM): 47.7 (42.1-55.3) ^e^ WM: 42.6 (37.7-47.5) CST: 42.8 (42.1-53.3) Control Group: 43.4 (37.4-49.5)	^b^Individual Rehab: 7.4 ±4.0 Group Rehab: 7.69±4.1 Control Group: 7.7±4.0	--	--
WOMAC Function Subscale Score^a^	^b^37.0 ± 13.2 ^c^ 53.7 ± 19.7	^b^ CST + Exercise: 33.1 ± 8.0 Control: 32.5 ± 8.3	^b^ Exercise: 34.3 ± 7.2 CST: 35.0 ± 7.4 CST + Exercise: 35.6 ± 7.3	--	^c^ 54.6 (17.1)	^c^ CBT: 53.0 (48.1−57.9) ^e^ Control Group: 48.4 (43.1−53.7)	--	^c^CST+ WM: 47.7 (42.2-53.1) ^e^ WM: 44.3 (39.5-49.0) CST: 46.2 (41.1-51.3) Control Group: 46.1 (39.7-52.5)	^b^Individual Rehab: 26.4±14.7 Group Rehab: 27.9 ±14.7 Control Group: 27.2±14.6	--	--
Arthritis Impact Measurement Scales 2- Pain Subscale^a^	--	--	--	Internet CST: 4.8 ± 1.7 Control Group: 5.1 ± 1.8	5.1 ± 2.1			CST+WM: 5.8 (5.3-6.2) WM: 5.3 (4.9–5.7) CST: 5.6 (5.2–6.0) Control Group: 5.5 (4.9–6.1)	--	CST: 5.4 ± 2.1 Spouse Assisted CST: 5.1 ± 1.7 Control Group: 5.2 ± 2.2	CST: 5.4 ± 2.1 Arthritis Education: 5.6 ± 1.9 Control Group: 5.8 ± 1.62
Arthritis Impact Measurement Scales 2 - Physical Function Subscale^a^	--	--	--	Internet CST: 1.7 ± 1.3 Control Group: 1.8 ± 1.0	1.8 ± 1.2	--	--	CST+WM: 1.7 (1.4–1.9) WM: 1.8: (1.1–1.7) CST: 1.6: (1.3–1.8) Control Group: 1.6 (1.2–1.9)	-	CST: 1.8 ± 0.9 Spouse Assisted CST: 1.7 ± 1.1 Control Group: 1.6 ± 0.7	CST: 2.0 ± 1.0 Arthritis Education: 2.1 ± 1.22 Control Group: 1.9 ± 1.2
Years with Arthritis Symptoms^a^	13.1 ± 10.0	CST + Exercise: 15% < 2 years, 51% 2-10 years, 34% >10 years Control: 32% < 2 years, 47% 2-10 years, 20% >10 years	Exercise: 6.0 (3-10)^d^ CST: 5.5 (4-10) CST + Exercise: 5.5 (2-10)	--	13.8 ± 9.9	CBT: 8.9 ± 8.7 Control Group: 6.6 ± 4.5	--	--	Individual Rehab: 7 (3–15) ^d^ Group Rehab: 5 (2.5–11) Control Group: 6 (3–15)	--	--
Number of Comorbidities^a^	8.5 ± 3.9	--	--	1.3 ± 1.1	--	CBT: 5.3 ± 2.8 Control Group: 4.9 ± 3.6	--	--	--	--	--
BMI, kg/m^2 a^	35.2 ± 8.2	31.4 ± 8.0	31.3 ± 6.1	--	33.4 ± 8.0	CBT: 30.1 ± 6.0 Control Group: 29.9 ± 6.3	--	CST+ WM: 34.1 (33.0–35.2) WM: 33.5 (32.4–34.7) CST: 34.4 (33.3–35.5) Control Group: 34.1 (32.8–35.4)	Individual Rehab: 30.0 (18–45) ^f^ Group Rehab: 30.18 (20–50) Control Group: 30.3 (20–51)	--	--
Health Behaviors and Coping Strategies											
Coping Skills Questionnaire – Total Coping Attempts^a^	93.9 ± 36.6	CST + Exercise: 61.7 ± 24.9 Control: 65.7 ± 24.9	--	--	--	--	--	--	--	CST: 69.2 ± 26.7 Spouse Assisted CST: 67.8 ± 23.2 Control Group: 58.9 ± 22.3	--
Coping Skills Questionnaire – Catastrophizing Subscale ^a^	11.4 ± 7.6	--	--	--	7.0 ± 6.4	--	--	CST+WM: 7.4 (5.5–9.3) WM: 6.6 (4.8–8.2) CST: 7.2 (5.5–8.9) Control Group: 6.9 (4.9–8.9)	--	--	--
Pain Catastrophizing Scale^a^	19.8 ± 12.3	CST + Exercise: 8.8 ± 9.2 Control: 10.1 ± 9.6	Exercise: 14.9 ± 8.1 CST: 14.8 ± 9.3 CST+ Exercise: 14.4 ± 9.7	--	--	CBT: 16.9 (13.8-20.0) ^e^ Control Group: 13.5 (10.7-16.2)	--		--	--	--
Arthritis Self-Efficacy Scale^a^	5.9 ± 2.0	--.	--	Internet CST: 6.7 ± 1.8 Control Group: 6.3 ± 2.1	6.0 ± 1.9	--	--	--	--	--	--

*CST* Coping Skills Training, *CBT* Cognitive Behavioral Therapy, *WOMAC* Western Ontario and McMaster Universities Osteoarthritis Index, *BMI* Body Mass Index

^a^Mean (standard deviation unless otherwise noted); ^b^WOMAC Likert scale version; ^c^WOMAC Visual Analog Scale or Likert scores transformed to 0-100 scale; ^d^Median (Interquartile Range); ^e^95% Confidence Intervals; ^f^ Mean (range)

### Sociocultural environment

#### Age

The mean age of STAART participants was 59 years (SD = 10.3), with a slightly lower age for VA participants than UNC participants. This mean age was slightly lower than other studies except for Somers et al. [25].

#### Sex

The proportion of females in the STAART study was 49%, which is lower than in other studies (range: 56–81%); among VA participants, only 21% were female, which reflects the high proportion of males in the VA.

#### Race and ethnicity

All STAART participants self-identified during screening as being black or African American, per study eligibility requirements. Two other studies included about 1/3 African Americans [25, 57], but the rest 13% or fewer (though several studies did not report information on race). Among STAART participants, 2.9% also self-identified as being of Hispanic or Latino ethnicity. Only Rini et al. reported ethnicity information for the sample, with 11% being of Hispanic or Latino ethnicity [57].

#### Education

Among STAART participants, 75% reported some education above high school, with the proportion being higher among VA than UNC participants. Proportions of participants with education above high school ranged from 61 to 86% in comparator studies.

#### Work status

Thirty-four percent of STAART participants reported they were currently working. Proportions of working participants ranged widely among other studies, from 21 to 57%.

#### Household financial status

Among STAART participants, about 1/3 reported that they could “just meet basic expenses” or “don’t have enough to meet basic expenses.” We did not identify any comparator studies that measured financial or income status in a manner that could be directly compared with the measure we collected for STAART participants.

#### Marital status

Forty-two percent of STAART participants were married or living with a partner as married, with a substantially higher proportion for VA than UNC (51% vs. 32%). In other studies, the proportions of married participants were all higher, ranging from 62 to 66%, though a number of studies did not report marital status. This comparison reflects a potentially greater need or risk for STAART participants.

#### Religiosity

Among STAART participants, mean scores on the DUREL were relatively high for all domains, including attendance at religious services, private religious activities and intrinsic religiosity. We did not identify any comparator studies that measured this construct.

### Biological vulnerability and mechanisms

#### Pain and function – WOMAC

The mean total WOMAC score (Likert version) among STAART participants was 53.0 (SD = 17.8), which indicates moderate to severe symptoms. WOMAC scores were somewhat worse for the VA group compared to the UNC group. One comparator study reported total WOMAC scores (Likert version) ranging from 38 to 39 [61]; this comparison indicates greater symptom severity among STAART participants. The mean WOMAC pain subscale score (Likert version) among STAART participants was 11.0 (SD = 3.9). Among three comparator studies that reported WOMAC pain scores on the Likert scale, mean values were all lower than for STAART participants (7.7–8.6); this comparison indicates greater pain among STAART participants [45, 55, 61]. When converted to a 0–100 scale, the mean WOMAC pain score among STAART participants was 55.0 (SD = 19.4); this score was worse than [25] or comparable to [59] comparator studies that used the WOMAC VAS version. The mean WOMAC function subscale score (Likert version) among STAART participants was 37.0 (SD = 13.2). Among three comparator studies that reported WOMAC function scores on the Likert scale, mean values were all lower (26–33); this comparison reflects poorer function among STAART participants [45, 55, 61]. When converted to a 0–100 scale, the mean WOMAC function score among STAART participants was 53.7 (SD = 19.7); this score was worse than [25] or comparable to [58, 59] other studies that measured the WOMAC VAS version.

#### Pain – AIMS

Several studies included AIMS or AIMS2 pain and function scores. Among these, AIMS pain scores ranged from 5.1–5.8 and AIMS2 pain scores ranged from 4.8–5.1; these scores represent modest levels of pain (scale range of 0–10). AIMS function scores ranged from 1.6–2.0 and AIMS2 function scores ranged from 1.7–1.8; these scores represent relatively low levels of functional limitations, potentially reflecting that these samples were less limited than participants in the STAART study.

#### Pain interference – PROMIS

Among STAART study participants, the mean score was 63.8 (SD = 6.9). This score indicates that the mean level of pain interference for STAART participants was a little over one standard deviation greater than the average of the general population. We did not identify any comparator studies that included this measure.

#### HRQoL – SF-12

The mean SF-12 PCS score for STAART participants was 33.1; this is lower than the average score for US men and women aged 60–69 (45.6 and 44.0, respectively), reflecting poorer HRQoL among STAART participants [63]. The mean SF-12 MCS score for STAART participants was 50.1 (SD = 11.1); this is slightly lower than the average score for US men and women aged 60–69 (52.7 and 51.8, respectively), also reflecting somewhat poorer HRQoL among STAART participants [63]. We did not identify any comparator studies that included this measure.

#### Duration of arthritis symptoms

On average, the self-reported duration of arthritis symptoms was 13.1 years (SD = 10.0), with a longer duration for VA participants than UNC participants. In comparator studies, duration of symptoms ranged from 5 to 14 years, with most having a mean duration lower than the STAART study.

#### Comorbid illnesses

The mean number of self-reported comorbid illnesses among STAART participants was 8.5 (SD = 3.9). Only two of the comparator studies reported a mean number of comorbidities for participants, and these were lower than in STAART (1.3–5.3), potentially indicating higher risk among our study sample [57, 59]. However, because of different comorbidity measures, the ability to directly to compare studies is limited.

#### BMI

The mean BMI among STAART participants was 35.2 kg/m^2^ (SD = 8.2), which corresponds to Class 2 (moderate risk) obesity; BMI was somewhat lower in the VA group compared to the UNC group. In comparator studies, mean BMI’s ranged from 30 to 34 kg/m^2^, indicating more risk among STAART participants.

### Health behaviors & coping strategies

#### Pain coping attempts – CSQ

Among STAART participants, the mean Pain Coping Attempts score was 93.9 (SD = 36.6), with slightly higher scores for UNC participants. Two other studies reported this scale [45, 46], with scores ranging from 59 to 69; this indicates that STAART participants were overall engaged in a larger number of coping efforts compared to other studies.

#### Pain catastrophizing – CSQ

The mean Pain Catastrophizing Scale score was 11.4 (SD = 7.6). This score was higher than two other studies reporting on this scale, in which mean scores ranged from 6.6–7.4 [25, 58]. This comparison suggests greater risk and needs for intervention among STAART participants.

#### Pain catastrophizing scale

The mean score on the PCS for STAART participants was 19.8 (SD = 12.3). Among three other studies reporting on this scale, mean scores ranged from 7 to 17 [45, 55, 59]. This comparison suggests greater risk and needs for intervention among STAART participants.

#### Depressive symptoms - PHQ-8

In the STAART study, the mean PHQ-8 score was 6.2 (SD = 5.3), and this was higher for VA participants than UNC participants. This average score indicates low depressive symptoms and is below the cutoff of 10 for depressive disorder [51]. We did not identify comparator studies that used this measure.

#### Arthritis self efficacy scale

The mean score for STAART participants on this measure was 5.9 (SD = 2.0). Two other studies administered the same version of this scale, and the mean score was similar to STAART participants [57, 58].

#### Self-efficacy for pain communication scale

The mean score for STAART participants was 78.7 (SD = 22.0); this score indicates a relatively low level of self-efficacy for communicating about pain [64]. We did not identify other comparator studies that used this measure.

#### Brief fear of movement scale

The mean score for STAART participants was 14.8 (SD = 3.5) on a scale of 6–24, suggesting a relatively high level of fear of movement. None of our comparator studies reported this measure.

## Discussion

With a focus on health disparities, the STAART study aimed to reach a patient group with greater OA severity and risk for other negative OA-related outcomes. The study particularly focused on African Americans, who have reported worse OA-related symptoms compared to Caucasians in several studies [2–4, 65, 66]. We also selected recruitment sites that serve many patients with multiple health challenges and relatively low income levels, since these individuals may be at particular risk for worse OA-related outcomes. Based on descriptions of comparator studies, none had a particular focus on identifying patient populations with greatest risks or needs. We utilized proactive and culturally tailored recruitment methods [8] and were able to meet the study sample size goal within the specified timeline, potentially reflecting a high degree of receptivity to this type of intervention in this patient group. Consented patients were slightly younger than those who declined or were ineligible, but there was a more pronounced gender difference, with the consented group including more females than those who declined or were ineligible. This may be due to greater willingness of women to engage in behavioral and psychological interventions; additional work is needed to understand how to best engage men in these types of programs.

Comparisons to similar studies of pain CST and CBT-informed pain coping strategies demonstrate that STAART participants differ in a number of factors that reflect worse OA severity and greater vulnerability for worsening outcomes, across all three domains we examined within the NIMHD Research Framework:

### Sociocultural environment

Based on our review of pain CST and or CBT-informed pain coping studies among patients with OA, STAART is the first to focus exclusively on African Americans. In most comparator studies, proportions of non-white participants were 10% or less, with none greater than about 1/3. This emphasizes the uniqueness and importance of the STAART study in adding to understanding of pain CST interventions among African Americans with OA.

STAART participants also differ from prior studies demographically in other ways that may affect pain-related outcomes and response to a pain CST intervention. Fewer STAART participants are married or living with a partner as married, compared with other studies, likely reflecting the lower rates of marriage among African Americans than Caucasians in general [67]; however, these rates may also partly reflect a lower income status among STAART participants, considering the clinics in which we recruited and the fact that marriage rates decline with lower income. This is an important difference from other studies, since close partners can offer support for pain coping, and being “un-partnered” can place individuals at greater risk for to other health-related or psychosocial stressors [23, 46]. To accommodate both married and single individuals and to reflect that pain communication goes beyond immediate support persons, the intervention encouraged participants to learn skills for communicating about pain with others more broadly, including family members, friends and health care providers. STAART participants, on average, are younger than samples of most prior studies in this area. This is likely due in part to higher risk of OA at younger ages among Veterans, who make up half of the STAART sample [68]. Although younger age is not necessarily a risk factor for worse pain-related outcomes, younger individuals with OA may be more likely to face challenges with continuing employment, particularly in physically demanding occupations. The STAART intervention is telephone-based, with a flexible schedule for calls, which may promote feasibility in working-age participants.

Although we were not able to directly compare financial status of participants across studies due to measurement inconsistency, about 1/3 of STAART participants perceived they “just meet” or “don’t even have enough to meet” basic expenses; as noted above, this may be partly reflect the underlying demographic characteristics of the clinics in which we recruited patients. This is important because financial stressors can augment the challenges of coping with chronic illnesses, and therefore individuals with lower income levels may particularly benefit from programs that teach and support coping skills. The telephone-based approach of STAART was also selected because it mitigates financial burdens related to transportation and missing work.

STAART participants reported relatively high levels of religiosity, which is important given the close connections between religiosity and multiple aspects of the pain and pain coping experience [69]. Unfortunately, we did not identify any comparator studies that measured this important construct. We expected that religious values would be important to a substantial proportion of STAART participants, and therefore one aspect of cultural tailoring involved encouraging participants to integrate elements of their spirituality or religious faith into their practice of pain coping when they felt it was important do so [8]. For example, during cognitive restructuring sessions, if participants identified that their faith played an integral role in reframing their pain-related challenges, this was further explored and built upon during the intervention.

### Biological vulnerability & mechanisms

Several key variables in this domain also indicate that STAART participants have greater risks and challenges than samples of comparator studies. First, the STAART sample overall had worse OA pain and function than participants in comparator studies. Although we could only make a direct comparison to studies that reported the same version of the WOMAC [45, 55, 61], indirect comparisons to studies using other versions of the WOMAC, as well as the AIMS, suggest STAART participants had worse symptoms [25, 46, 57–59, 62]. The mean total WOMAC score for the STAART sample reflects moderate-to-severe symptoms. We expect this difference from other studies reflects worse OA symptoms among African Americans than Caucasians, which has been shown in a number of prior studies [2–4].

The STAART sample also had longer symptom duration than most comparator studies [55, 59, 61]. Although it is not clear whether the effectiveness of cognitive behavioral interventions differs based on time since symptom onset, it is an important consideration that overall, this group of patients had been managing their chronic pain for a longer period of time than patients in prior studies of this type. This difference from other studies may partly reflect a younger age of onset of OA among some military personnel and Veterans [68]. BMI was higher in our study than in any of the comparators, including Somers et al. [25], which selected only overweight and obese individuals. This is likely a reflection of higher BMI among African Americans in the US, compared with Caucasians [70]. Although it was difficult to compare comorbidity burden to other studies, STAART participants had a high number of comorbid illnesses (mean of 8.5). This likely reflects the high prevalence of multiple chronic health conditions (e.g. hypertension, diabetes, cardiovascular disease) among African Americans [70], but as noted above, this also may be partly attributable to the general patient populations of the clinics in which we recruited. Little is known about the associations of comorbidity with pain coping in OA, but the challenge of managing multiple health conditions may increase the difficulty of coping with OA-related symptoms.

### Health behaviors & coping strategies

STAART participants also differed from prior studies in ways that may indicate greater need for a pain CST intervention. First STAART participants reported higher levels of pain catastrophizing than any other study [25, 45, 46, 55, 58, 59]; prior research has also found higher levels of catastrophizing among African Americans than Caucasians [11–13]. Pain catastrophizing can be improved by CST interventions, which emphasize cognitive restructuring as a strategy to address unhelpful thoughts about pain [20, 22, 24]. Participants in the STAART study also reported more coping attempts than other studies that measured this same construct [45, 46]. This may be due in part to the higher levels of pain experienced by STAART participants compared with prior study samples.

STAART participants reported relatively low levels of Self-Efficacy for Pain Communication. Although none of our comparator studies measured this construct, STAART participants’ scores were similar to those of another sample of individuals with OA [64]. Based on prior work by Campbell et al. [71], we expected that many patients would benefit from building skills and confidence in communicating with others about their pain experience; therefore, a pain communication module was included [8]. Fear of movement scores also were high among STAART participants. Although the STAART CST intervention does not specifically address fear of movement, other modules (e.g. activity pacing, cognitive restructuring) involve related concepts and have potential to reduce pain-related fear of movement. STAART participants had relatively low depressive symptoms, based on the mean PHQ-8 score. Although none of the comparator measures used this same measures, some studies measured depressive symptoms with other measures, including the Beck Depression Inventory, Depression Anxiety Stress Scales, Hospital Anxiety and Depression Scale, and the Geriatric Depression Scale [55, 58–61]; participants in these studies also had scores that indicated normal or low levels of depressive symptoms, similar to the STAART study.

## Conclusion

In conclusion, this comparison of STAART participants to prior studies of CST for OA identified differences in a number of key variables related to OA severity and risks for poor pain-related outcomes. In particular, STAART participants have worse OA symptoms, higher BMI, and greater levels of pain catastrophizing compared to other study samples. STAART participants also have a high comorbidity burden, and 1/3 perceive they have relatively low income. These characteristics place STAART participants at greater risk for worse OA-related physical and psychological outcomes. However, pain CST programs can improve multiple OA-related outcomes, and STAART participants may gain particular benefit from this intervention approach because of its focus on pain catastrophizing. If results of the STAART study support the effectiveness of pain CST in this group, this will be an important addition to prior literature, given the importance of identifying effective interventions for African Americans, who bear a higher burden of OA.

## Additional file

Additional file 1: Participant Inclusion criteria and recruitment methods for comparator studies. (DOCX 26 kb)

## Abbreviations

AIMS: Arthritis impact measurement scales
AIMS2-SF: AIMS2 Short Form
BMI: Body mass index
BWM: Behavioral weight management
CBT: Cognitive behavioral therapy
CSQ: Coping strategies questionnaire
CST: Coping skills training
DSM-IV: Diagnostic and statistics manual fourth edition
DUREL: Duke University Religion Index
HRQoL: Health-Related Quality of Life
MCS: Mental health composite score
NIMHD: National Institute on Minority Health and Health Disparities
OA: Osteoarthritis
PCS: Pain catastrophizing scale
PCS: Physical health composite score
PCST: Pain coping skills training
PHQ-8: Patient Health Questionnaire 8
SD: Standard deviation
SF-12: Short-Form-12
STAART: Pain Coping Skills Training for African Americans with OsteoaRTthritis
UNC: University of North Carolina at Chapel Hill
VA: Durham VA Healthcare System
VAS: Visual analog scale
WOMAC: Western Ontario and McMasters Universities Osteoarthritis Index
