# Supplementary material for: Pain coping skills training for African Americans with osteoarthritis study: baseline participant characteristics and comparison to prior studies
Source: BMC Musculoskelet Disord. 2018 Sep 19;19:337. doi: 10.1186/s12891-018-2249-6 (PMC6145122; doi:10.1186/s12891-018-2249-6)
Supplement: Supplementary file 1 — Participant Inclusion criteria and recruitment methods for comparator studies. (DOCX 26 kb) [file 12891_2018_2249_MOESM1_ESM.docx]

**Additional file 1: Participant Inclusion Criteria and Recruitment Methods for Comparator Studies.**

**Effectiveness of an Internet-Delivered Exercise and Pain-Coping Skills Training Intervention for Persons With Chronic Knee Pain: A Randomized Trial (Bennell et al., 2017)**

Participants: 148 patients from Australia who had knee pain during walking (score of >=4 on an 11-point numerical rating scale in the previous week), mild to moderate physical dysfunction (score>20 out of 68 on the physical function subscale of the WOMAC), and knee pain for more than 3 months and on most days of the previous month.

Recruitment methods: Community participants were recruited via print, social media and radio advertisements.

**Physical Therapist-Delivered Pain Coping Skills Training and Exercise for Knee Osteoarthritis: Randomized Controlled Trial (Bennell et al., 2016)**

Participants: 222 patients with radiographic evidence of knee OA from Melbourne and Brisbane, Australia. Participants had pain for >= 3months, at least moderate difficulty with daily activities (based on WOMAC physical function subscale >=25 of 68 units) and average pain during previous week >=40 on 100 mm VAS.

Recruitment methods: Recruitment strategies included advertisements in various community sources, brochures in clinical settings, presentations about the study, and a database of individuals from prior studies.

**Automated Internet-based pain coping skills training to manage osteoarthritis pain: a randomized controlled trial (Rini et al., 2015)**

Participants: 113 patients with clinically confirmed OA in one or both knees and/or hips, from a rural geographical region in North Carolina and Duke University Medical Center.

Recruitment methods: Participants were recruited for screening from the Johnston County Osteoarthritis Project (2) or through medical or research records from Duke University Medical Center.

**Nurse practitioners can effectively deliver pain coping skills training to osteoarthritis patients with chronic pain: A randomized, controlled trial (Broderick et al., 2014)**

Participants: 256 patients with chronic pain caused by OA of the knee or hip from New York, Virginia and North Carolina. Participants had a physician confirmed diagnosis of knee or hip OA, and usual pain >=4 on a 10-point scale for at least 6 months.

Recruitment methods: Participants were recruited from community primary care and rheumatology practices. Advertisements containing study information were posted in practice waiting rooms, and participating doctors informed eligible patients about the opportunity to participate in the study during regular office visits.

**Effectiveness of a cognitive-behavioural group intervention for knee osteoarthritis pain: a randomized controlled trial (Helminen et al., 2014)**

Participants: 111 patients with radiographic evidence, symptomatic knee OA in a medium sized city in Finland. Participants had knee pain most days for at least a month, rated as >=40 on a 100mm on the VAS version of the WOMAC.

Recruitment methods: Letters were sent to patients age 35-75 in primary care locations who had knee radiographs indicating OA.

**Cognitive-behavioral treatment for comorbid insomnia and osteoarthritis pain in primary care: the lifestyles randomized controlled trial (Vitiello et al., 2013)**

Participants: 367 patients with clinically significant OA pain and insomnia who received care for OA at Group Health in Washington state. Significant OA pain was defined as grade II, III or IV pain on the Graded Chronic Pain Scale.

Recruitment methods: Participants were initially screened for eligibility via a mailed survey and then contacted by phone.

**Pain coping skills training and lifestyle behavioral weight management in patients with knee osteoarthritis: a randomized controlled study (Somers et al., 2012)**

Participants: 232 patients with radiographic OA in one or both knees from Duke University Medical Center. Other inclusion criteria included: knee pain on most days of the month for at least 6 months, overweight or obese with a BMI of >=25 and <=42, no other major weight bearing joint affected by OA and OA of the knee(s) considered to be the medical condition contributing most to limitations in daily functions.

Recruitment methods: Participants were recruited from advertisements in local newspapers, flyers posted in the community or via physical referral.

**Clinical effectiveness of a rehabilitation program integrating exercise, self-management, and active coping strategies for chronic knee pain: a cluster randomized trial (Hurley et al., 2007)**

Participants: 418 patients in inner city primary care practices who had consulted a physician for mild, moderate or severe knee pain of > six-months duration.

Recruitment methods: Participants were identified through database searches of the 54 consenting practices. Interested participants contacted the study team by phone.

**Spouse-assisted coping skills training in the management of osteoarthritic knee pain (Keefe et al., 1996)**

Participants: 88 married patients with knee OA from Duke University Medical Center.

**Pain coping skills training in the management of osteoarthritic knee pain: A comparative study (Keefe et al., 1990)**

Participants: 99 patients with knee OA from Duke University Medical Center.
